# Hepatic-to-azygos vein redirection after a failed bifurcated graft Fontan conversion

**DOI:** 10.1093/icvts/ivad050

**Published:** 2023-04-05

**Authors:** Junichi Koizumi, Akio Ikai, Tomoyuki Iwase, Hajime Kin

**Affiliations:** Department of Cardiovascular Surgery, Iwate Medical University, Morioka, Japan; Department of Cardiovascular Surgery, Shizuoka Children’s Hospital, Shizuoka, Japan; Department of Pediatric Cardiac Surgery, Al Jalila Children’s Hospital, Dubai, United Arab Emirates; Department of Cardiovascular Surgery, Iwate Medical University, Morioka, Japan

**Keywords:** Pulmonary arterio-venous fistula, Absent inferior vena cava, Azygos vein

## Abstract

A successful hepatic-to-azygos vein redirection was performed in a patient with absent inferior vena cava using a long vascular graft to address a pulmonary arterio-venous fistula after a failed Fontan conversion. No exacerbation was observed 5 years postoperatively.

## INTRODUCTION

Pulmonary arterio-venous fistula (PAVF) is a well-known complication in patients with absent inferior vena cava (IVC) [1]. Lack of hepatic vein (HV) flow in the pulmonary circulation can lead to PAVF [2]. HV redirection procedure is often challenging in anatomically complex cases. We report a case of successful HV-azygos vein (AzV) redirection using a long graft.

## CASE REPORT

**Figure 1:**
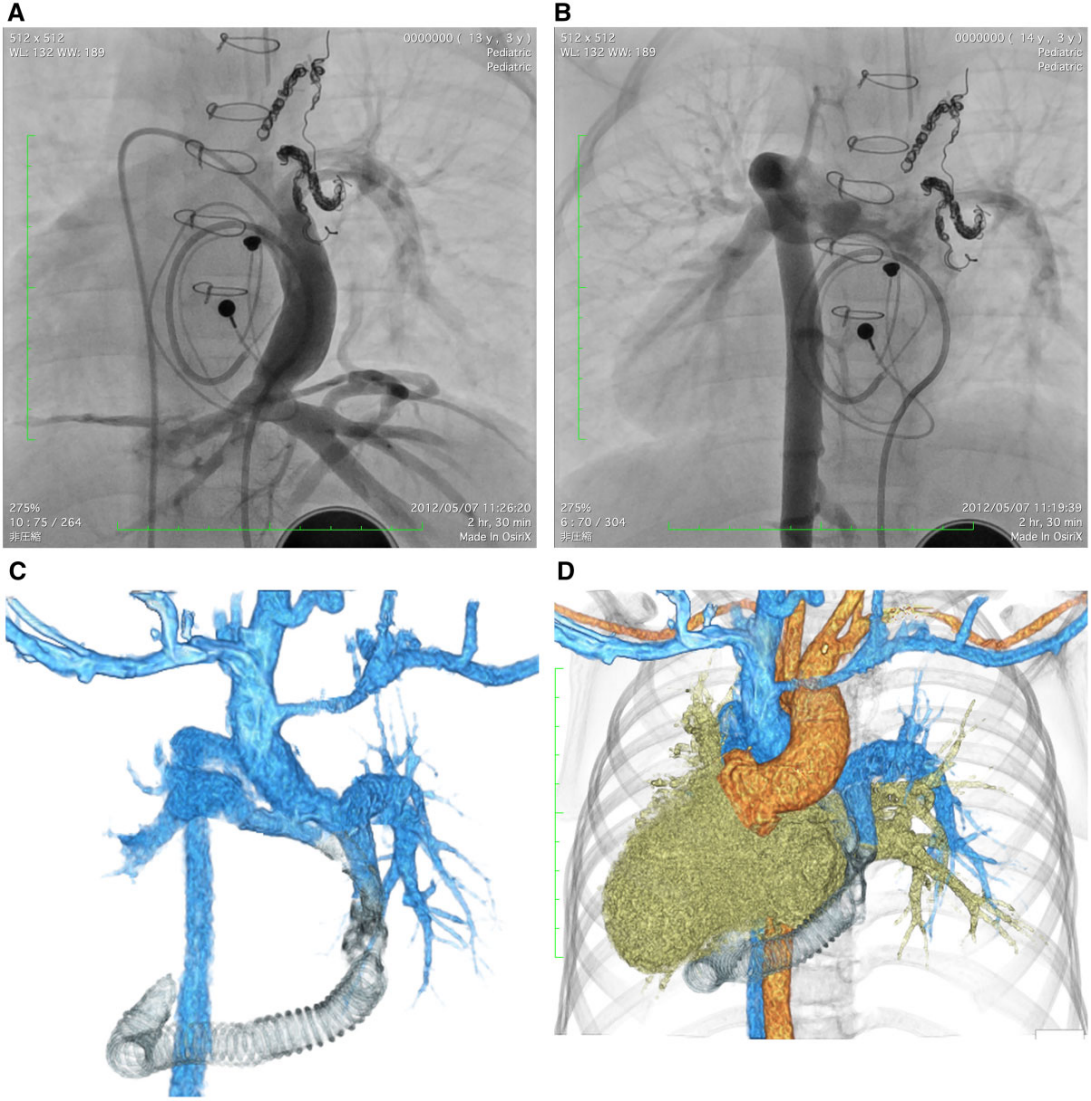
(**A** and **B**) Angiography findings after the hepatic vein-to-pulmonary artery conduit procedure. (**C** and **D**) Computed tomography findings after the hepatic-to-azygos vein redirection.

Herein, we present the case of a 9-year-old female with polysplenia, dextrocardia, a single ventricle, pulmonary atresia, right-sided superior vena cava (SVC) and absent IVC with azygos continuation. She underwent a modified Blalock shunt and pacemaker implantation at 1 month of age. Subsequently, she had a Kawashima procedure at 8 months of age, and an extracardiac HV–pulmonary artery (PA) conduit procedure using a 16-mm graft was conducted at 17 months of age. Her oxygen saturation increased from 85% to 95% after the procedure; however, it decreased to 90% 2 years postoperatively. Catheterization (Fig. 1A and B and Supplementary Material, Fig. S1) and contrast echocardiogram revealed the left dominant HV flow and the right PAVF without pulmonary stenosis or major aortopulmonary collaterals. We performed coil embolization of the venovenous collaterals from the innominate vein (Supplementary Material, Fig. S2); however, her oxygen saturation did not improve. At 3 years of age, the patient underwent a Fontan conversion using an 18 mm × 9 mm bifurcated graft but failed with right leg occlusion in the follow-up. To improve the PAVF, HV-AzV redirection was scheduled at the age of 9. The patient’s guardian provided informed consent.

**Figure 2:**
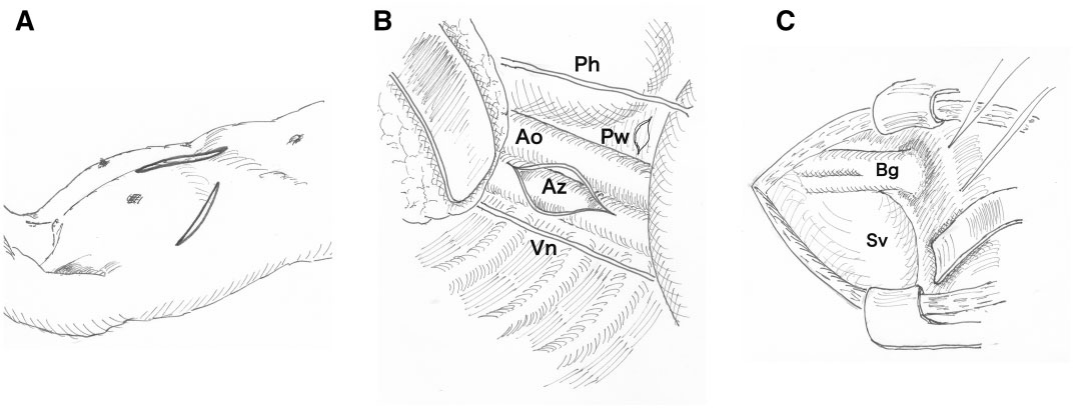
(**A**–**C**) Surgical scheme. Ao: descending aorta; Az: azygos vein; Bg: bifurcated graft; Ph: phrenic nerve; Pw: pericardial window; Sv: single ventricle; Vn: vagal nerve.

The patient was placed in the left hemi-decubitus position. A right anterolateral thoracotomy at the 7th intercostal space and a lower-half sternotomy was performed (Fig. 2A–C). The AzV was partially clamped and anastomosed with a 10-mm expanded polytetrafluoroethylene ringed graft. The graft was introduced in the pericardial window below the phrenic nerve. The proximal portion of the bifurcated graft was partially clamped and anastomosed with the graft. The length of the graft resulted in about 10 cm. Finally, both legs were ligated (Video 1). A cardiopulmonary bypass was not used. Aspirin and warfarin were administered before and after the procedure. Computed tomography (Fig. 1C and D) and angiography (Video 2) showed the HV-AzV graft and adequate hepatic venous flow. Her oxygen saturation was 92% at the 5-year follow-up without exacerbations (Supplementary Material, Table S1).

## DISCUSSION

HV flow distribution is often unpredictable after an HV inclusion procedure in patients with absent IVC due to the low flow rate of HV and sensitivity to small offset between SVC and HV conduits [3]. In contrast, the HV-AzV connection seems to be the most reliable method to establish a balanced HV flow [4]. Furthermore, the HV-AzV connection sometimes presented with better energy preservation than a classical HV–pulmonary artery conduit [5].

To avoid an HV-preferential flow leading to PAVF, we believe that an HV-AzV connection should be considered foremost if the anatomical situation allows it. In a case with a contralateral dominant SVC and cardiac apex, the AzV can be easily accessed through posterior pericardiotomy. Contrarily, in a case with ipsilateral dominant SVC, as in this case report, all HV inclusion procedures carry challenges and risks due to the ventricular mass. We concluded that an HV-AzV redirection procedure using a long graft was another option for anatomically complex cases despite the higher risk of thrombosis. We believe that a smaller graft should be used to prevent the stagnation of blood, as the low flow rate of the HV may lead to thrombus formation.

## CONCLUSION

HV-AzV redirection is a reliable method to achieve a balanced HV flow distribution regardless of the anatomical variations in patients with absent IVC.

## Supplementary Material

ivad050_Supplementary_Data

## Data Availability

The data underlying this article are available in the article and its online supplementary material. Interdisciplinary CardioVascular and Thoracic Surgery thanks Katarzyna Januszewska and the other anonymous reviewer(s) for their contribution to the peer review process of this article.
