# Supplementary material for: Hepatic-to-azygos vein redirection after a failed bifurcated graft Fontan conversion
Source: Interdiscip Cardiovasc Thorac Surg. 2023 Apr 5;36(4):ivad050. doi: 10.1093/icvts/ivad050 (PMC10120161; doi:10.1093/icvts/ivad050)
Supplement: ivad050_Supplementary_Data [file ivad050_Supplementary_Data.zip › Supplementary Table S1.docx]

Supplementary Table S1: Oxygen saturation at different stages.

| Stage | Oxygen saturation (%) on room air | Age |
| --- | --- | --- |
| Pre-HV-PA conduit | 85 | 17 months old |
| Post-HV-PA conduit | 95 | 17 months old |
| Pre-bifurcated graft | 90 | 3 years old |
| Post-closure VV collateral | 90 | 3 years old |
| Post- bifurcated graft | 90 | 3 years old |
| Pre-HV-AzV redirection | 87 | 9 years old |
| Post- HV-AzV redirection | 89 | 9 years old |
| Current status | 92 | 14 years old |
