# Supplementary figures and images for: Hepatic-to-azygos vein redirection after a failed bifurcated graft Fontan conversion
Source: Interdiscip Cardiovasc Thorac Surg. 2023 Apr 5;36(4):ivad050. doi: 10.1093/icvts/ivad050 (PMC10120161; doi:10.1093/icvts/ivad050)

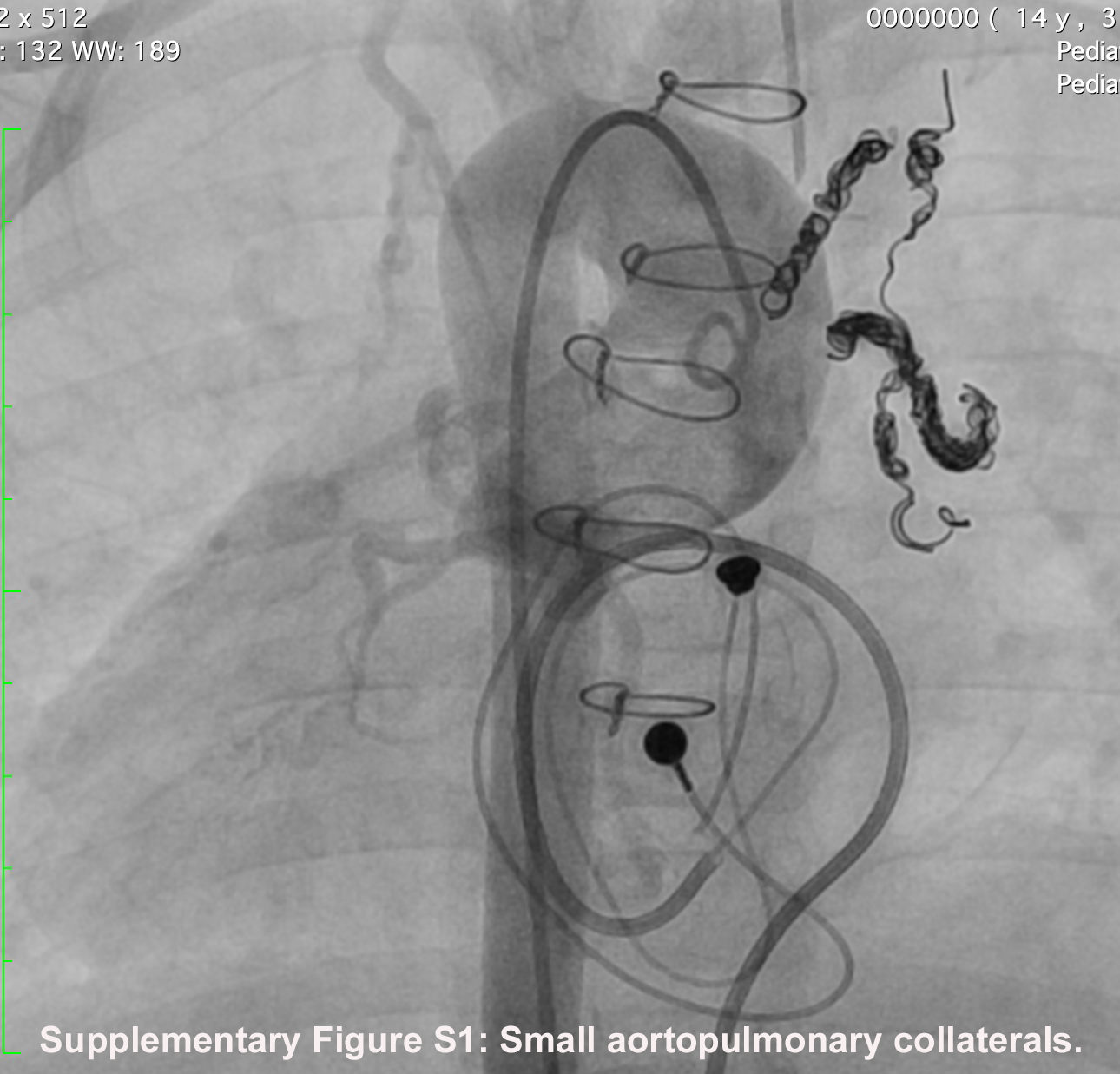

Supplement: ivad050_Supplementary_Data [file ivad050_Supplementary_Data.zip › Suppl Fig S1 AoG post HV-PA.tif]

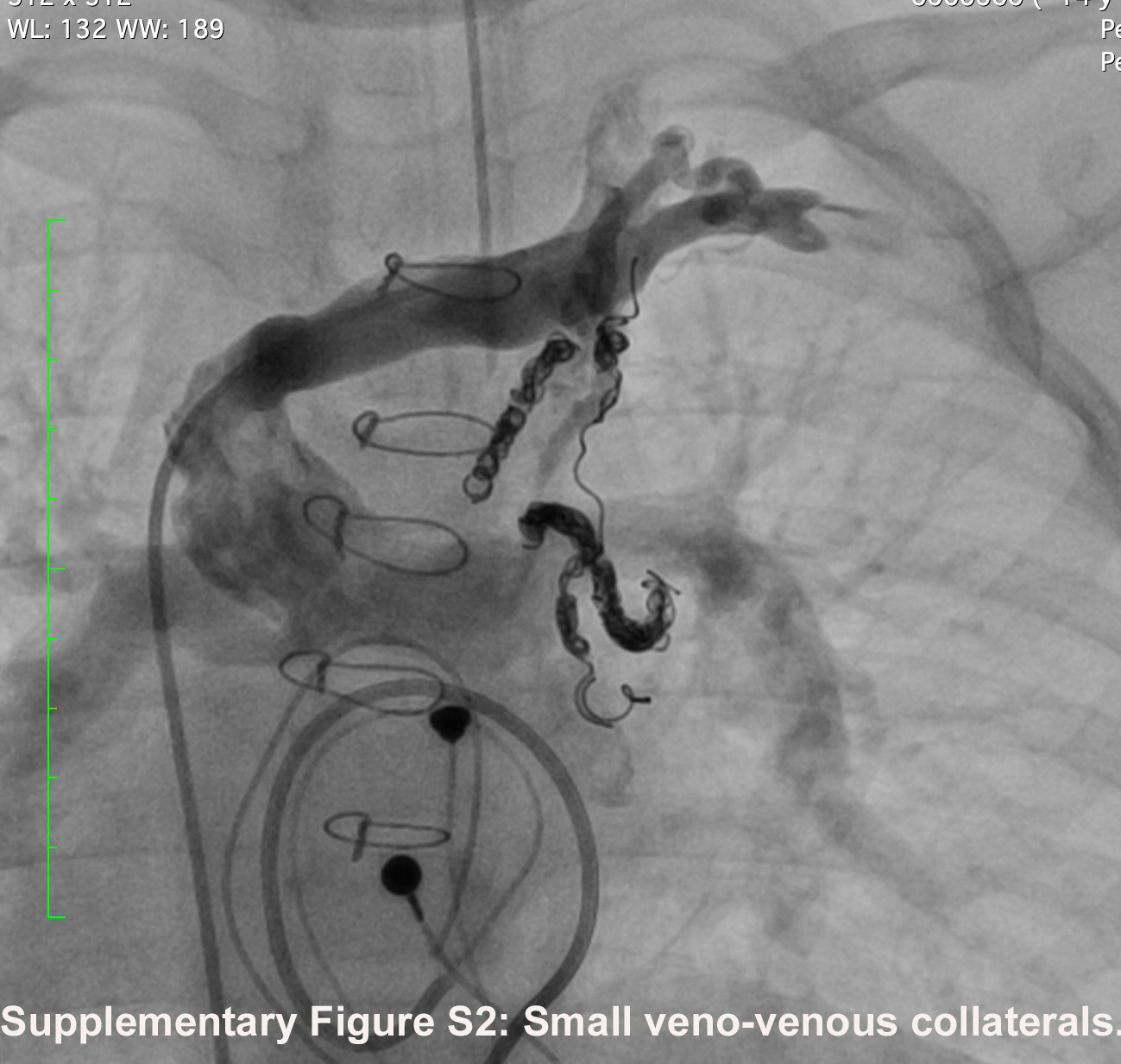

Supplement: ivad050_Supplementary_Data [file ivad050_Supplementary_Data.zip › Suppl Fig S2 INNV VV collat.tif]
